# Studying the Suitability of Nineteen Lignins as Partial Polyol Replacement in Rigid Polyurethane/Polyisocyanurate Foam

**DOI:** 10.3390/molecules27082535

**Published:** 2022-04-14

**Authors:** Christián Henry, Akash Gondaliya, Mark Thies, Mojgan Nejad

**Affiliations:** 1 Department of Forestry, Michigan State University, 480 Wilson Road, East Lansing, MI 48824, USA; henrych5@msu.edu; 2 Department of Chemical Engineering and Materials Science, Michigan State University, East Lansing, MI 48824, USA; gondaliy@msu.edu; 3 Department of Chemical and Biomolecular Engineering, Clemson University, Clemson, SC 29634, USA; mcths@clemson.edu

**Keywords:** lignin, polyisocyanurate, polyurethane, rigid foam, biobased, low-density, thermal insulation, polyol

## Abstract

In this study, nineteen unmodified lignins from various sources (hardwood, softwood, wheat straw, and corn stover) and isolation processes (kraft, soda, organosolv, sulfite, and enzymatic hydrolysis) were used to replace 30 wt.% of petroleum-based polyol in rigid polyurethane/polyisocyanurate (PUR/PIR) foam formulations. Lignin samples were characterized by measuring their ash content, hydroxyl content (Phosphorus Nuclear Magnetic Resonance Spectroscopy), impurities (Inductively Coupled Plasma), and pH. After foam formulation, properties of lignin-based foams were evaluated and compared with a control foam (with no lignin) via cell morphology, closed-cell content, compression strength, apparent density, thermal conductivity, and color analysis. Lignin-based foams passed all measured standard specifications required by ASTM International C1029-15 for type 1 rigid insulation foams, except for three foams. These three foams had poor compressive strengths, significantly larger cell sizes, darker color, lower closed-cell contents, and slower foaming times. The foam made with corn stover enzymatic hydrolysis lignin showed no significant difference from the control foam in terms of compressive strength and outperformed all other lignin-based foams due to its higher aliphatic and *p*-hydroxyphenyl hydroxyl contents. Lignin-based foams that passed all required performance testing were made with lignins having higher pH, potassium, sodium, calcium, magnesium, and aliphatic/*p*-hydroxyphenyl hydroxyl group contents than those that failed.

## 1. Introduction

Rigid polyurethane foams are widely used in structural and insulative applications because of their combination of good adhesion to various substrates, low-density to high-compression strength ratio, and high thermal insulation properties [1]. Polyurethane (PUR) and polyisocyanurate (PIR) foams are the two main types of isocyanate-based rigid foams using urethane chemistry [2]. Rigid polyurethane foams are created by the step-growth polymerization reaction of polyisocyanates and polyols in the presence of additives, including catalysts, surfactants, and blowing agents. Polyisocyanurate foams are prepared by reacting an excess of isocyanate (200–350 isocyanate index [1]), polyol, and additives, including trimerization catalysts such as amines, bases, and metal oxides [3]. Due to stricter worldwide fire regulations, polyisocyanurate foams have become more popular since their commercialization in 1996 [1,2]. Trimerization of isocyanate in polyisocyanurate foams results in improved fire performance and decreased smoke generation compared to polyurethane foam [4,5,6]. Because pure polyisocyanurate foam creates brittle products, a mixture of polyurethane and polyisocyanurate (PUR/PIR) is used for most applications [7].

The increase in polyurethane foam consumption, fluctuations in the price of polyurethane raw materials, and the toxicity of the raw materials used for manufacturing polyols (e.g., propylene oxide) all have pushed researchers to find greener alternatives [8,9]. Lignin, the most abundant natural aromatic polymer on earth, has excellent potential to replace petrochemical polyols due to its hydroxyl functional groups that can react with isocyanate to form polyurethane linkages [10,11,12,13]. Lignin is a byproduct of chemical pulping and bioethanol production, and only 2–5% is used in value-added products [14,15,16]. The renewable fuel standard program announced that 60 billion gallons of biofuel will be produced by 2030 [17], which will further increase the supply of lignin, encouraging lignin valorization [18]. The addition of lignin into polyurethanes has been reported to improve antioxidant [19], antimicrobial [20], fire resistance [21], and biodegradability properties [22].

Unmodified, lignin-based rigid polyurethane (PUR) foams have been successfully formulated by replacing up to 30 wt.% of the petrochemical polyol, with the majority of the work focusing on high-density foams (>60 kg/m^3^) [22,23,24,25,26]. Luo et al. [22] incorporated 0–25% lignin, dispersed in soy polyol for 3 h, into high-density rigid PUR foam. They reported that incorporating lignin decreased foam density while improving biodegradability, as well as the mechanical and thermal properties of the foams [22]. Pan and Saddler [25] compared the performance of high-density rigid PUR foams made by substituting 19–30% of a commercial polyol with organosolv and kraft hardwood lignins. They found that foams made with hardwood organosolv had higher compressive strength than the foams made with hardwood kraft lignin due to better miscibility of the organosolv lignin in the petroleum-based polyol. Compared to the control foam without lignin, they reported that incorporation of both lignins decreased foam density and compression strength [25]. Liu et al. [24] replaced 15 wt.% of a petroleum-based polyol with refined alkali lignin in the formulation of high-density rigid PUR foam and reported that lignin-based foams had lower apparent density and thermal conductivity than the control foam. The incorporation of unmodified lignin in low-density rigid PUR foam significantly increased polyol viscosity over 6.3 wt.% lignin loading (high-density foams were produced above 4.3 wt.%) [27]. At 1.2 wt.% lignin loading, the maximum compression strength of lignin-based foam was greater than the control. However, compression strengths of lignin-based foams decreased over 2 wt.% lignin loading while foam densities increased [27]. Xue et al. [23] reported that compression strength and density of lignin-based low-density polyurethane foams decreased with lignin addition (high-density foams were made up to 16% lignin loading and lignin loading above 24% created low-density foam). Low-density lignin-based rigid PUR foams did not meet compression strength requirements for insulative applications [23].

A knowledge gap exists in using lignins in low-density polyurethane/polyisocyanurate (PUR/PIR) rigid foam applications. Previously, sorbitol [4], tannin [5], crude glycerol, algae, and castor oil [28] have been used to formulate low-density PUR/PIR foams at various polyol replacement percentages (10–100%). This is the first study to focus on partially replacing petrochemical polyol in low-density PUR/PIR rigid foam with unmodified lignin while comparing the suitability of a wide range of unmodified, mostly industrially isolated lignins. As the molecular structure of lignin varies considerably based on both source and isolation method [25,29,30], it is crucial to characterize lignin samples to determine their differences and choose the most suitable lignin for a specific application. Utilizing a wide variety of unmodified lignin to prepare low-density rigid PUR/PIR foams will help elucidate the best lignin for PUR/PIR foam applications.

In this study, nineteen unmodified lignin samples were analyzed and used to replace 30 wt.% of petrochemical polyol in rigid PUR/PIR foam formulations. Thirty percent polyol substitution was chosen based on previous works in the literature that reported a decline in foam properties with higher than 30 wt.% loading of unmodified lignin [23,31]. The objective of this research was to determine the most suitable lignin for rigid PUR/PIR foam applications and study the correlations between lignin properties and foam performance.

## 2. Results and Discussion

### 2.1. Lignin Characterization

The ash and impurity analysis results of lignin samples are important parameters to consider because high cation levels are known to catalyze both polyurethane (PUR) and polyisocyanurate (PIR) reactions in PUR/PIR foams [1], increasing compression and fire properties. However, asynchronous gelling/blowing reactions [32] and too much PIR formation as a result of isocyanate trimerization are known to create brittle foams [7]. Transition metals, strong bases, alkali metal alkoxides, carboxylic acid salts, acetates, carbonates, carboxylates (K, Na, Ca, Mg, etc.), and various organometallic compounds can also catalyze the isocyanate trimerization (PIR) reaction [1,3,7]. The maximum acceptable level of potassium and sodium in rigid PUR foams using petrochemical polyols is around 100 ppm or 0.01% [1]. Ideally, higher cation content (impurities) of lignins would not be as detrimental for PIR/PUR foams and could increase the reactivity of solid lignins with isocyanate. Impurity analysis results in Table 1 show that the majority of lignins had potassium and sodium contents, over the acceptable limit for PUR foams (>0.01% [1]).

The pH of lignin samples (Table 1) ranged from 3.7–7.9 and showed no significant difference based on the lignin source or isolation process. Overall, kraft lignins had the highest average pH (5.4 ± 1.3), followed by enzymatic hydrolysis (5.0 ± 0.2), organosolv (4.6 ± 1.1), soda (4.4 ± 0.3), and lignosulfonate (4.3 ± 0.1) lignins. Maillard et al. [33] reported that lignin’s pH has a significant effect on the reaction time of lignin with isocyanate when used in flexible polyurethane foam. They used kraft lignin with pHs ranging from 2.4–6.6 and observed that foam reaction time decreased with increasing pH [33]. The decrease in reactivity with acidic lignins and isocyanate is likely due to the neutralization of polyurethane catalysts [33].

Hydroxyl content is mostly determined using titration for industrial polyurethane applications [34], but since the dark color of lignin makes it difficult to determine the titration point of the polyol, phosphorus nuclear magnetic resonance spectroscopy (^31^P NMR) was utilized in this study. The percent weight replacement of lignin (30 wt.% of polyol) was used in this study instead of the molar ratio/hydroxyl value replacement of polyol (to calculate isocyanate) to better compare the effect of various lignins on foam performance. Using weight percent substitution instead of equivalent weight/molar ratio allowed us to study the impact of lignin properties (i.e., ash, impurities, and hydroxyl contents) on foam performance. All lignins were within the acceptable hydroxyl value range (>200 mg KOH/g or equal to 3.56 Total OH content in mmol/g) [1,7] for rigid polyurethane foam production (Table 2). It is important to note that the 3.56 minimum does not take individual hydroxyl group types and ratios into account, just the total hydroxyl content. Therefore, more work needs to be conducted to determine the exact amount or ratio of each type of hydroxyl group to create optimal foams. Lignin 5-SW-L was insoluble in ^31^P NMR solvents, so we were unable to measure its hydroxyl value. Lignin 1-CS-E, enzymatic corn stover lignin, had the highest (*p* < 0.05) aliphatic (3.41 mmol/g), p-hydroxyphenyl (1.16 mmol/g), and total hydroxyl (7.84 mmol/g) contents of all lignins. Hydroxyl contents of lignins were significantly different based on the lignin source and isolation process (*p* < 0.05).

### 2.2. Foam Characterization

**Figure 1:**
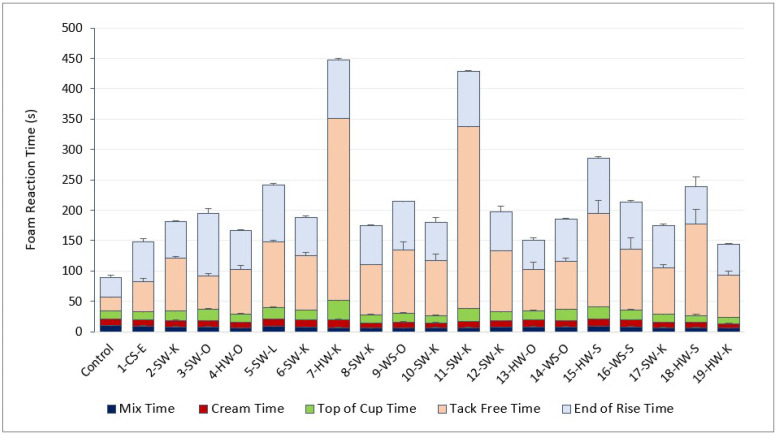
Foam reaction time in seconds.

Figure 1 shows foam reaction times, including mix, cream, top of the cup, tack-free, and end of rise times. These reactivity measurements indicate various reactions, including the onset of the isocyanate and water reaction (mix time), the isocyanate and polyol reaction (cream time), blowing activity (top of cup time), foam curing (tack-free time), and lastly, the foam reaction rate (end of rise and total reaction times) [1,35]. It is crucial to control the timing of these reactions to create optimal foam properties such as density, compression strength, and cell size [7].

The mix, cream, top of the cup, and end of rise times of lignin-based foams were comparable to the control foam (with no lignin), and statistically, there was no significant difference between them. However, the tack-free time of lignin-based foams, indicating the curing times of foams, were significantly higher (>129%) than the control foam. The higher tack-free times increased the total reaction time of lignin-based foams, making total reaction times of lignin-based foams about 60–400% higher than the control foam without lignin. This result was expected due to the incorporation of solid lignin and has also been observed by previous researchers when incorporating lignin into rigid PUR foam [26,27].

On average, the total reaction times for all lignin-based foams were 157% higher than the control, indicating that the reactivity of the foams decreased with the addition of lignin. Since lignin was in a solid-state, unlike the commercial polyol (liquid-state), decreased reactivity was anticipated. The increase in lignin-based foam reaction times is also due to the steric hindrance effect of lignin hydroxyl groups, which decreases their reactivity with isocyanate [26,36,37]. It has also been reported that the addition of lignin increases the viscosity of the polyol blend mixture, which also affects foam reactivity by reducing the mobility of raw materials in the solution [26,38].

**Figure 2:**
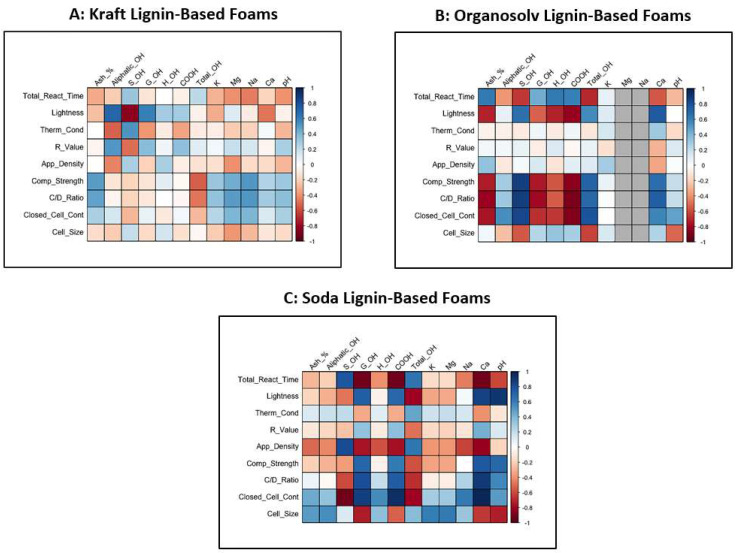
Heat map of correlations (r ranges from 1 to −1) between lignin properties vs. lignin-based foam performance based on (**A**) kraft lignin-based foams, (**B**) organosolv lignin-based foams, (**C**) soda lignin-based foams. Values above ± 0.7 show a high correlation.

The total reaction time of kraft, soda, and organosolv lignin-based foams negatively correlated with lignin pH, r = −0.4, −0.6, and −0.3, respectively (Figure 2). Overall, lignin-based foams made with more alkaline lignins (higher pH) had faster reaction times than lignins with lower pH (r = −0.3). This is because acidic/low pH can neutralize polyurethane catalysts, increasing reaction time. For example, since the kraft isolation process utilizes aqueous sodium hydroxide and various sulfides, the higher pH can lead to phenolic hydroxyl group ionization, increasing lignin solubility (in co-polyol) [39]. This increased solubility of lignin in co-polyol could also improve the reactivity of lignin (reduced reaction time) [25].

**Figure 3:**
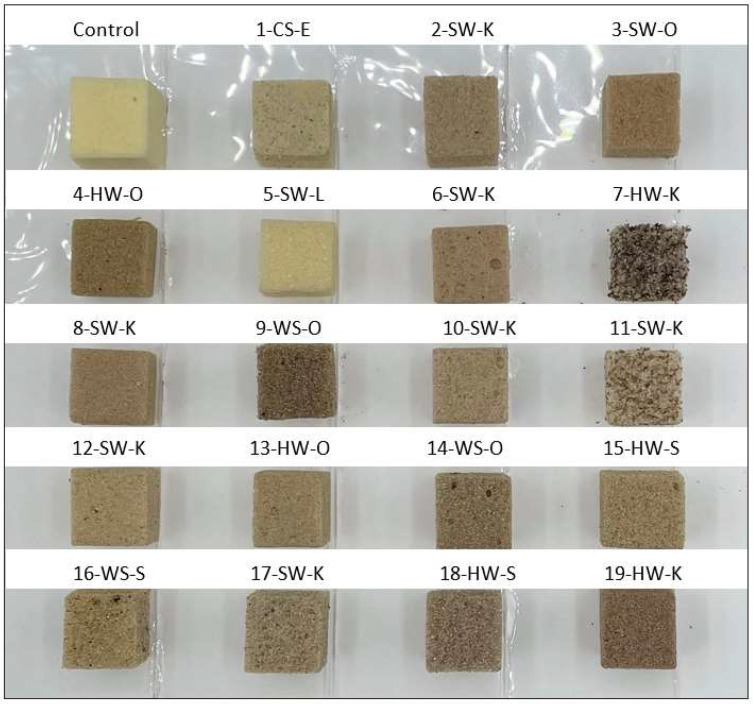
Images of lignin-based (30 wt.%) and control foams. Samples 3-SW-O, 7-HW-K, and 11-SW-K failed the compression strength and/or closed-cell content tests.

Lignin-based foams were 2–50% darker than the control foam with no lignin (measured by spectrophotometer) shown in Table 3. This can also be observed in Figure 3, which shows all the prepared control and lignin-based foam samples. Foam brittleness and lignin agglomeration can be observed in samples 7-HW-K and 11-SW-K, which affected various foam properties (discussed later). The average foam lightness (the higher the L value, the whiter or lighter the foam) of lignosulfonate foam (78) was significantly higher than enzymatic hydrolysis (69), kraft (57), soda (57), and organosolv (49) lignin-based foams. Though lignin-based foams are darker than the control, the color of foam is not an issue, as the foams would be hidden behind wall or ceiling panels when used for insulation applications.

**Figure 4:**
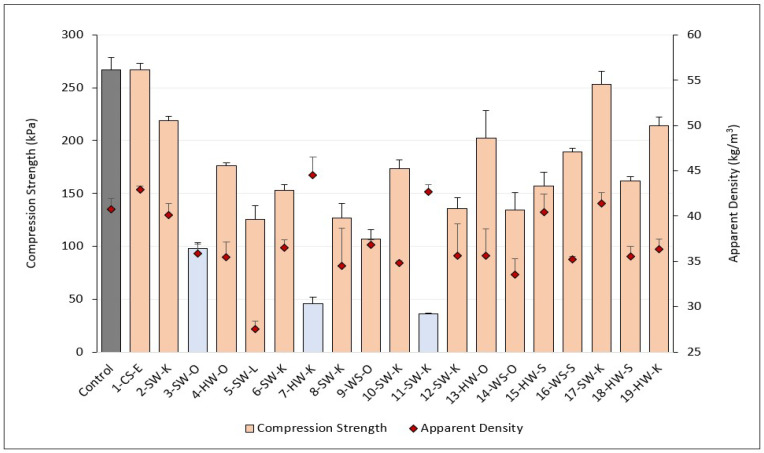
Compression strength and apparent density of foams. Blue bars failed the compression test (<104 kPa). Note that the axis does not start at 0 for apparent density.

With respect to density (Figure 4), the lignin-based foams (except 5-SW-L) showed no significant difference from the control and were within the acceptable 30–60 kg/m^3^ range for low-density rigid polyurethane foams. Foams made with enzymatic hydrolysis lignin had an average density (43 kg/m^3^) that was significantly higher than foams made with kraft (38 kg/m^3^), soda (37 kg/m^3^), organosolv (35 kg/m^3^), and lignosulfonate (28 kg/m^3^) lignins. Compared to control foams (Figure 4), on average, lignin-based foams had 13% lower densities. The decrease in density with lignin addition has been reported in other lignin-based rigid PUR foam studies at 1–15% lignin loading [24,27], but to the best of our knowledge, this is the first time this has been reported in low-density lignin-based rigid PUR/PIR foam.

Interestingly, foams made with lignins 7HW-K and 11-SW-K had higher densities than the control foam. Their significantly higher reaction times (447 and 488 s, respectively) compared to other lignin-based foams (<285 s) can explain their increased density compared to the control foam (as is evident from their photos in Figure 3). These two lignins are likely acting as fillers. Moreover, their significantly slower gelation reaction rates lead to more blowing agent loss, which can create foam with higher densities [26,43]. On the other hand, the small increased density of foams made with lignins 1-CS-E and 17-SW-K is likely due to the increased reactivities in these lignins, creating thicker cell walls and more crosslinking within the foams [44], which was also confirmed by our compression strength results shown in (Figure 4).

**Figure 5:**
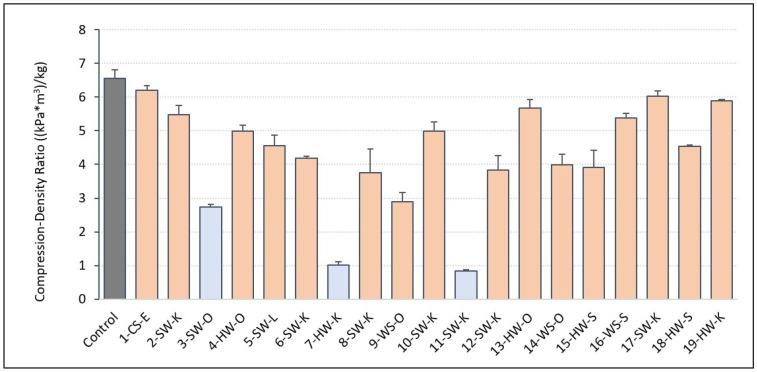
Compression–density ratio (CDR) of formulated foams. Foams in blue failed in compression testing.

Compression strength is one of the most important properties of rigid foams because it can be a predictor of other foam properties such as volumetric change during the service life of foams [45]. All but three lignin-based foams (3-SW-O, 7-HW-K, and 11-SW-K) met or exceeded the 104 kPa minimum compressive strength requirement for type 1 rigid foams [41]. Eight lignin-based foams also met more stringent compression strength requirements for type 2 rigid foams of 173 kPa (1, 2, 4, 10, 13, 16, 17, and 19). Lignin-based foams that passed the minimum compression strength requirement (orange bars in Figure 4) had significantly lower reaction times, apparent density, cell size, and thermal conductivity than those that failed (blue bars in Figure 4 and Figure 5).

The compression strength of lignin-based foams was negatively correlated (r = −0.7) with foam reaction times, meaning lignins with faster reaction times made better foams. In general, the addition of lignin into PUR/PIR foams decreased compression strength compared to the control foam with no lignin. This decrease in compression strength of lignin-based foams is likely due to solid lignin particles in the foam matrix causing decreased reactivity and increasing polyol mixture viscosity, creating more irregular cell sizes in the final foam [26,38]. The average compression strength of foam made with 1-CS-E (267 kPa) showed no significant difference from the control foam (267 kPa) made with petroleum-based polyol and was significantly higher than foams made with soda (169 kPa), kraft (150 kPa), organosolv (144 kPa), and lignosulfonate (126 kPa) lignins. Compared to other lignin-based foams, the increased compression strength of foam made with 1-CS-E lignin is likely due to the higher aliphatic and *p*-hydroxyphenyl hydroxyl contents (Table 2) of corn stover lignin, creating more crosslinking within the foam and increasing foam reactivity, density, and compression strength (Figure 1 and Figure 4).

Since each foam was not formulated with the same density, we calculated the foam compression to density ratio (CDR) shown in Figure 5. Given that all foams (excluding lignin 5-SW-L) fell within the low-density range of 30–60 kg/m^3^ for polyurethane foams, the effect of density is not significant [7]. This was also proven by our correlation data, finding no correlation between apparent density and compression strength of formulated foams (r = 0.04). Foams with CDR values < 2.9 did not meet the minimum required compression strength of 104 kPa for the spray insulation applications. CDR was found to have high correlation with closed-cell content (r = 0.8), total reaction time (r = −0.8), and cell size (r = −0.6) of lignin-based foams.

**Figure 6:**
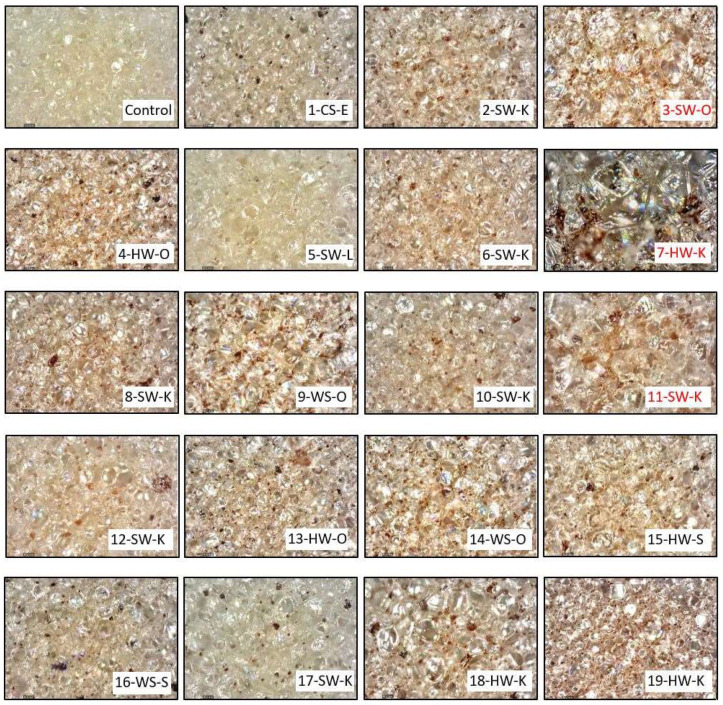
Digital microscope images of formulated foams using 160× magnification. Lignin-based foams 3-SW-O, 7-HW-K, 11-SW-K failed in compression testing.

Cell morphology, i.e., cell size/shape and closed-cell content, is essential in producing rigid polyurethane foams with consistent performance. Smaller and more uniform cells make the foam act as a homogenous material with similar properties throughout [1], whereas larger cells hold more blowing agents, decreasing the thermal conductivity of foam. As seen in Table 3, lignin-based foams that passed the compression test had cell sizes comparable to the control (~6% smaller). Figure 6 shows digital microscope images of formulated foams used to measure foam cell size. Foams that failed compression testing (3-SW-O, 7-HW-K, and 11-SW-K) showed more visual irregularity and had significantly larger (31%) cell sizes than those that passed (*p* < 0.05). The increase in cell size for these lignin-based foams can be explained by their higher reaction times than the control formulation (Figure 1). The cell size of lignin-based foams was found to correlate with foam total reaction time (r = 0.8) and closed-cell content (r = −0.8).

Closed cells (>90% content) are crucial in maintaining the thermal conductivity properties of formulated rigid foams. High closed-cell content ensures less blowing agent loss and creates foams with lower thermal conductivities. All foams except 7-HW-K and 11-SW-K passed the minimum 90% closed-cell requirement for rigid foams (Table 3). Lignin-based foams that failed in compression strength testing (3-SW-O, 7-HW-K, and 11-SW-K) had significantly lower closed-cell contents than lignin-based foams that passed (86 and 99%, respectively). Closed-cell content of lignin-based foams was found to correlate with the total reaction time of foam (r = −0.9), CDR (r = 0.8), and foam compression strength (r = 0.7). Lignin-based foams that passed the minimum requirement for closed-cell content showed comparable results (within 2%) to the control formulation.

Thermal conductivity consists of λgas, λradiation, λsolid, and λconvection, with λgas being the main component [1]. Higher-density foams have higher λsolid and therefore higher thermal conductivity. Because the majority of thermal conductivity comes from λgas, our foams were within the low-density range of 30–60 kg/m^3^, and all foams were made with the same blowing agent, there was no significant difference between the thermal conductivities of control and lignin-based foams. All foams were significantly below the maximum (257 mW/mK) acceptable thermal conductivity (Table 3) in rigid insulation foams (type 1–4) [41] and had high R values, making them suitable for insulation applications.

The high impurity content of lignin (Table 1) and the solid state of lignin are likely why other researchers have reported issues with polyol viscosity, foam cell structure, and other foam properties with lignin loadings over 30% in rigid polyurethane foam [23,31]. Strong correlations between lignin-based foam properties and impurity content were most prominent in soda and organosolv lignin-based foams (Figure 2). Specifically, calcium (organosolv and soda) and sodium (soda) affected foam properties such as apparent density, compression strength, closed-cell content, and cell size. To the best of our knowledge, this is the first study to measure the effect of lignin impurities on lignin-based polyurethane/polyisocyanurate foam properties (effects on rigid polyurethane foams have not been studied either). Since these impurities would likely have a greater impact at higher lignin loading percentages, more in-depth work needs to be conducted to study the effect of lignin impurity content on rigid polyurethane and polyurethane/polyisocyanurate foam properties.

## 3. Materials and Methods

### 3.1. Materials

Commercial lignin samples from hardwood (HW), softwood (SW), corn stover (CS), and wheat straw (WS) sources and from enzymatic hydrolysis (E), sulfite or lignosulfonate (L), soda (S), organosolv (O), and kraft (K) processes were purchased from or provided by lignin producers and used without further modification. Huntsman LLC. (The Woodlands, TX, USA) graciously provided foam raw materials. As shown in Table 4, formulations included polymeric methylene diphenyl diisocyanate (pMDI), polyol (polyester-based), surfactant (polyalkylene), catalysts (amine), blowing agents (water and n-pentane), viscosity reducer, and phosphate-based flame retardant. Other reagents for lignin characterization, including tetrahydrofuran (THF), pyridine, and acetic anhydride, were purchased from Fisher Scientific (Waltham, MA, USA) and were used as received without any further purification.

### 3.2. Lignin Characterization Methods

Each lignin sample was sieved using an 80 µm mesh sieve to reduce the effect of lignin particle size on foam properties. The samples were then oven-dried (80 °C) until a constant weight was achieved to ensure no moisture remained in the lignin to react with isocyanate.

#### 3.2.1. Ash Content

The percent ash content of lignin samples was determined gravimetrically following TAPPI T 211 om-02 [46]. Firstly, ceramic crucibles were oven-dried at 105 °C to a constant weight, cooled in a desiccator, and weighed (nearest 0.1 mg). Two grams of lignin were then loaded into each crucible, oven-dried at 105 °C to a constant weight, cooled, and weighed. The crucibles were then placed in a Thermolyne Furnatrol muffle furnace (Fisher Scientific, Waltham, MA, USA) and heated with a 5 °C/minute ramp to 525 °C with a 4 h dwell time. Samples were then cooled to 100 °C, transferred to a desiccator, and weighed. Percent ash was then determined from the weight of ash and of oven-dried lignin.

#### 3.2.2. Impurity Analysis

To determine the effect of impurities, the amount of calcium, sodium, magnesium, and potassium in lignin samples were measured (A&I Great Lakes Laboratories, Fort Wayne, IN, USA). Lignin samples were prepared following the 922.02 [47] and 980.03 [48] methods developed by the Association of Analytical Chemists (AOAC). In brief, samples were ground using a Wiley Mill (#10 sieve) and oven-dried at 105 °C overnight. After preparation, lignin samples (0.2 g each) were open vessel microwave digested (MARS 5, CEM Corp, Matthews, NC, USA) following SW 846-3051A [49] in a two-step process. First, samples were diluted with 2 mL of nitric acid, heated up to 90 °C, and held at that temperature for 90 s. Second, the solution was cooled to 50 °C; then, 1 mL of peroxide was added, and the solution was heated to 105 °C and held at that temperature for 10 min. The samples were cooled and brought to a final dilution volume of 25 mL with peroxide and analyzed using an inductively coupled plasma optical emission spectrometer (ICP-OES), Thermo Scientific iCAP 6000 series 6500 Duo, according to AOAC 985.01 [50]. Multi-element standards from Inorganic Ventures (Christiansburg, VA, USA) along with a blank sample were used for calibration.

#### 3.2.3. pH Measurements

The pH of lignin samples was determined in at least triplicate by adding 0.1 g lignin in 10 mL distilled water and stirring for 5 min at 350 rpm. Stirring was stopped 20–30 s before measuring the pH on a Fisher brand Mettler Toledo SevenCompact pH/Ion meter (Columbus, OH, USA).

#### 3.2.4. Hydroxyl Content

The hydroxyl content of each lignin was determined using phosphorus nuclear magnetic resonance (^31^P NMR) spectroscopy following previously published methods [51,52,53]. First, 40 mg lignin was dissolved in a solvent solution (325 μL, 1.6:1 *v*/*v*) of pyridine and deuterated chloroform along with 300 μL of dimethylformamide (DMF). Then 100 μL of cyclohexanol solution (22 mg/mL in anhydrous pyridine and deuterated chloroform, 1.6:1, *v*/*v*) was added as an internal standard. In the next step, 50 μL of chromium (III) acetylacetonate solution (5.8 mg/mL in anhydrous pyridine and deuterated chloroform, 1.6:1, *v*/*v*) as relaxation reagent was added to the mixture in anhydrous pyridine and deuterated chloroform (1.6:1, *v*/*v*). Lastly, 100 μL of 2-chloro-4,4,5,5-tetramethyl-1,3,2-dioxaphospholane was added as phosphitylation reagent. The spectra were acquired using an Agilent DDR2 500 MHz NMR spectrometer (Billerica, MA, USA) equipped with 7600AS, running VnmrJ 3.2 A. Data were obtained using a 5 mm tube (600 μL solution), a 90° pulse angle flip with a relaxation delay of 5 s, and 128 scans. Hydroxyl values were then calculated for each lignin by multiplying the total hydroxyl content in mmol/g by 56.1 (mass of KOH) [1,53].

### 3.3. Foam Preparation Methods

All foam samples were formulated in accordance with the standard practice for polyurethane raw materials: polyurethane foam cup test ASTM D7487-13 [35] using a 237 mL cup. B-side polyol blends were prepared by mixing specified amounts of polyol, lignin, catalysts, surfactant, and blowing agent in a predetermined ratio (Table 1) for thirty seconds using an overhead digital high-speed mixer (3000 rpm). Isocyanate was added to the polyol blend and mixed until the heat was felt on the outside of the cup.

Foam reaction time was determined by measuring various characteristics, including mix, cream, top of the cup, tack-free, and end of rise times [11,54]. These measurements allow for the prediction of mixture reaction time and foam performance in industrial applications [7], specifically spray polyurethane foams. In brief, the timer was started after isocyanate was poured into B-side components for 3 s. The first measurement, “mix time”, was taken once the heat was felt on the outside of the cup, showing the onset of the isocyanate and water reaction. Cream time was measured when the mixture turned to a creamy color and began to rise. The top of the cup (time) was recorded once the foam reached the top of the cup, and tack-free time was noted when the skin/surface of the foam could be touched without sticking. End of rise time was taken when the foam stopped rising, signaling the end of major reactions. Foams with tack-free times higher than three minutes were assigned a value of 300 s.

### 3.4. Foam Characterization Methods

Foam properties were measured at least 72 h after formulation. After 24 h, samples were cut to size using a razor blade and a tabletop band saw (Grizzly G0803Z). After cutting, samples were stored at room temperature for at least 48 h before each analysis.

#### 3.4.1. Foam Lightness

Foam lightness was determined using the CIEL*a*b* color system as measured with a Konica Minolta CM-2300d spectrophotometer (Ramsey, NJ, USA) using the L* measurement (L* or lightness ranges from 0–100 with 0 being the darkest and 100 being the lightest). Three measurements were taken on each foam sample 25 mm below the top of the cut foam (perpendicular to the foam rise direction) and averaged.

#### 3.4.2. Apparent Density

The apparent density of formulated foams was determined according to ASTM D1622 [55]. Twenty-five-millimeter cube samples were measured volumetrically to 0.01 mm using a digital caliper. Each side was measured three times and averaged. The weight of each cube was measured using a digital scale (0.0001 g). Averages of at least five foam samples were used to calculate the density reported in kg/m^3^. Foams were not reformulated to have the same density in order to allow us to measure the effect of lignin properties. The effect of density has been reported as insignificant when rigid foams are within the range of 30–60 kg/m^3^ [7].

#### 3.4.3. Compression Strength

The compression strength of each foam sample was determined via ASTM D1621-16 [56] using an Instron 5565 universal testing machine (Norwood, MA, USA). Twenty-five-millimeter cube samples were tested perpendicular to the foam rise in at least triplicate using a 2.5 mm/min strain rate until specimens were 13% of their initial thickness. Compression strength was recorded for each sample as the maximum compressive stress divided by the initial cross-sectional area of each specimen.

#### 3.4.4. Cell Size

The average cell size (diameter) of each foam sample was measured using a Dino-Lite Edge digital microscope (Torrance, CA, USA) following a modified version of ASTM D3576-15 [40]. Three-millimeter slices were cut from 25 mm below the top of each foam. Cell size was calculated by averaging the size of at least twenty cells (160× magnification) from formulated foams perpendicular to the foam rise.

#### 3.4.5. Closed-Cell Content

Closed-cell content was determined according to ASTM D6226-15 [57]. A micromeritics gas pycnometer (AccuPyc II 1340, Norcross, GA, USA) under nitrogen atmosphere was used to perform the analysis following micromeritics method B [58]. Analysis conditions included 10 purges and 10 cycles along with 27.58 kPa purge and cycle fill pressures at 0.03 kPa/min. First, the foam resin density was found by grinding 12 g of foam and running the sample using the above conditions. The resin density was then entered into the pycnometer to begin closed-cell content analysis. Two 25 mm cube samples were weighed, run, cut three times, and run again. Cutting the sample three times doubled the number of cuts, allowing the pycnometer to correct for the cells that were open due to cutting and actual open-cell content.

#### 3.4.6. Thermal Conductivity

Thermal conductivity is the most important property of rigid foams used for insulation application and is usually measured via steady-state heat transfer [1,59,60]. This method is not always feasible for lab-scale foaming due to large sample requirements (0.3 m × 0.3 m × 0.05 m) [60]. The following needle probe technique has been reported to have accuracy within 5% and has been widely used in liquid and solid material for thermal conductivity measurements [60,61]. In our study, the thermal conductivity of the foams was determined using a Meter TEMPOS TPA machine (Pullman, WA, USA). Measurements were taken in the center of the foams, perpendicular to foam rise (in at least triplicate) using a 60 mm KS-3 probe. In order to convert thermal conductivity to R Value, the reciprocal of the probe length in meters (0.06 m) divided by the thermal conductivity was taken.

### 3.5. Statistical Analysis

SAS software (Cary, NC, USA) running the GLM procedure was used to compare lignin and foam data. Lignin-based foams were grouped based on pass/fail in compression strength tests and compared to the control foam with no lignin. Tukey HSD was used for mean separation (*p* < 0.05) between lignin (source and process), lignin-based foam (pass/fail), and control foam data. Outliers were determined using the 1.5 IQR rule. Pearson’s correlation was used to assess correlations between lignin and lignin-based foam properties. To obtain more specific correlation data between lignin and foam properties, data were grouped/separated by process and then rerun. Heat maps were created using R Studio: openxlsx, graphics, plyr, corrplot, pairwise.complete.obs, and correlmatcom.

## 4. Conclusions

This is the first study to partially replace petroleum-based polyol with nineteen lignins from various sources and isolation processes in low-density rigid polyurethane/polyisocyanurate (PUR/PIR) foam formulations. Lignins with higher aliphatic and *p*-hydroxyphenyl contents, higher pH, and higher sodium, calcium, magnesium, and potassium contents performed well in foam preparation and testing, demonstrating that the properties of lignin have a profound impact on foam performance regardless of lignin source and isolation process. Except for three, all the other lignin-based foams met or exceeded required performance based on ASTM standards for insulation spray foams. The lignin-based foams that failed the compression test had significantly lower reactivity, higher density, cell size, and thermal conductivity than those lignin-based foams that passed. However, the three lignins that did not pass testing could still be used in flexible foams or other applications where compression strength and closed-cell content are not as critical. Among tested lignins, the biorefinery corn stover lignin was found to be the most suitable lignin for partially replacing petroleum-based polyols in formulating PUR/PIR rigid foam due to its significantly higher aliphatic and *p*-hydroxyphenyl hydroxyl content. The results of this study will allow lignin producers to optimize their lignins for rigid foam production, foam producers to select the best lignins for foam application and scale-up, and, last but not least, expand the body of knowledge for lignin and lignin-based rigid PUR/PIR foams.

## Figures and Tables

**Table 1:** Ash, pH, K, Mg, Na, and Ca content of lignin samples.

Label	Ash Content (%)	pH	K (%)	Mg (%)	Na (%)	Ca (%)
1-CS-E	0.63 ± 0.01	4.99 ± 0.16	0.01	0.00	0.03	0.01
2-SW-K	3.92 ± 0.35	6.65 ± 0.11	0.12	0.01	0.68	0.04
3-SW-O	0.37 ± 0.11	5.04 ± 0.58	0.01	0.00	0.01	0.01
4-HW-O	0.13 ± 0.03	6.42 ± 0.44	0.01	0.00	0.01	0.01
5-SW-L	11.5 ± 0.18 *	4.35 ± 0.07	0.22	0.17	0.11	3.81
6-SW-K	1.99 ± 0.20	3.97 ± 0.09	0.16	0.00	0.95	0.01
7-HW-K	1.11 ± 0.07	4.49 ± 0.26	0.06	0.01	0.18	0.06
8-SW-K	0.01 ± 0.01	6.64 ± 0.09	0.04	0.02	0.28	0.05
9-WS-O	0.50 ± 0.17	3.69 ± 0.10	0.01	0.00	0.01	0.01
10-SW-K	0.54 ± 0.02	4.83 ± 0.19	0.02	0.01	0.06	0.03
11-SW-K	0.65 ± 0.01	4.80 ± 0.17	0.02	0.00	0.17	0.01
12-SW-K	0.76 ± 0.02	4.90 ± 0.10	0.02	0.01	0.20	0.01
13-HW-O	0.04 ± 0.02	4.02 ± 0.07	0.02	0.00	0.01	0.03
14-WS-O	0.09 ± 0.03	3.88 ± 0.03	0.01	0.00	0.01	0.02
15-HW-S	0.11 ± 0.05	4.23 ± 0.15	0.15	0.01	0.76	0.17
16-WS-S	0.86 ± 0.15	4.71 ± 0.17	0.00	0.00	0.37	0.02
17-SW-K	0.94 ± 0.01	4.28 ± 0.09	0.03	0.02	0.40	0.04
18-HW-S	4.84 ± 0.09	4.17 ± 0.17	0.86	0.01	0.89	0.02
19-HW-K	5.19 ± 0.01	7.94 ± 0.04	0.28	0.02	1.12	0.17

* Significantly different *p* < 0.05. CS = corn stover, SW = softwood, HW = hardwood, WS = wheat straw, E = enzymatic hydrolysis, K = kraft, O = organosolv, S = soda, and L = lignosulfonate.

**Table 2:** Hydroxyl content determination results of lignin samples.

Label	Hydroxyl Content ^31^P NMR Data (mmol/g)							Hydroxyl Value
	Aliphatic	Syringyl	Condensed Phenolic	Guaiacyl	*p*-Hydroxy Phenyl	Carboxylic	Total OH	(mg KOH/g)
1-CS-E	3.41 *	0.74	0.39	1.08	1.16 *	1.06	7.84 *	439
2-SW-K	1.98	-	1.09	1.9	0.24	0.45	5.66	318
3-SW-O	1.04	-	0.47	1.57	0.18	0.47	3.73	209
4-HW-O	1.38	1.44	0.43	0.77	0.17	0.32	4.51	253
6-SW-K	2.10	-	1.31	2.82	0.25	0.68	7.16	402
7-HW-K	1.09	2.47	0.63	1.03	0.17	0.34	5.73	321
8-SW-K	2.08	-	1.29	2.13	0.18	0.59	6.27	352
9-WS-O	0.72	0.79	0.38	0.99	0.35	0.42	3.65	205
10-SW-K	2.07	-	1.29	2.16	0.19	0.54	6.25	351
11-SW-K	1.78	-	0.91	2.09	0.27	0.45	5.50	309
12-SW-K	2.49	-	1.43	2.26	0.24	0.37	6.79	381
13-HW-O	0.92	1.79	0.89	0.68	0.12	0.28	4.68	263
14-WS-O	1.12	0.69	0.24	0.87	0.34	0.41	3.67	206
15-HW-S	1.92	0.42	1.01	2.22	0.29	0.77	6.63	372
16-WS-S	1.36	1.24	0.42	1.04	0.25	1.18	5.49	308
17-SW-K	1.51	-	0.62	1.68	0.21	0.39	4.41	247
18-HW-S	1.80	0.68	0.31	0.64	0.42	1.03	4.88	274
19-HW-K	1.52	1.93	0.65	0.97	0.13	0.21	5.41	304

* Significantly different *p* < 0.05. Lignin 5-SW-L was not soluble; thus, we were unable to analyze it.

**Table 3:** Measured properties of formulated foams.

Label	Lightness (Color Analysis)	Cell Size (mm)	Closed-Cell Content (%)	Thermal Conductivity (mW/mK)	R-Value (Km^2^/W at 0.06 m)
Control	81 ± 0.5	0.22 ± 0.02	98.6 ± 0.03	9.2 ± 0.7	6.5 ± 0.6
1-CS-E	69 ± 0.7	0.23 ± 0.01	99.0 ± 0.05	9.1 ± 0.3	6.6 ± 0.2
2-SW-K	61 ± 0.6	0.22 ± 0.01	99.6 ± 0.05	10.1 ± 0.2	6.0 ± 0.1
3-SW-O	47 ± 0.5	0.22 ± 0.01	97.2 ± 0.01	9.9 ± 0.2	6.1 ± 0.1
4-HW-O	49 ± 0.3	0.15 ± 0.01	99.1 ± 0.02	9.4 ± 0.1	6.4 ± 0.1
5-SW-L	78 ± 0.2	0.26 ± 0.02	97.3 ± 0.02	9.2 ± 1.0	6.6 ± 0.8
6-SW-K	58 ± 1.5	0.16 ± 0.02	99.1 ± 0.13	9.8 ± 0.4	6.1 ± 0.2
7-HW-K	41 ± 0.7	0.32 ± 0.02	83.1 ± 0.14	12.9 ± 0.7	4.7 ± 0.3
8-SW-K	61 ± 0.8	0.20 ± 0.03	98.4 ± 0.05	9.0 ± 0.5	6.7 ± 0.4
9-WS-O	45 ± 0.4	0.19 ± 0.02	96.9 ± 0.06	9.4 ± 0.6	6.4 ± 0.5
10-SW-K	64 ± 0.9	0.11 ± 0.02	99.6 ± 0.04	9.3 ± 0.2	6.4 ± 0.1
11-SW-K	54 ± 1.4	0.39 ± 0.04	77.0 ± 0.04	10.9 ± 1.8	5.7 ± 1.0
12-SW-K	67 ± 1.7	0.20 ± 0.00	98.2 ± 0.05	10.2 ± 0.3	5.9 ± 0.2
13-HW-O	55 ± 1.1	0.19 ± 0.03	99.0 ± 0.14	10.0 ± 0.4	6.0 ± 0.3
14-WS-O	48 ± 1.0	0.25 ± 0.03	97.8 ± 0.04	10.1 ± 1.0	6.0 ± 0.5
15-HW-S	58 ± 1.7	0.19 ± 0.02	98.5 ± 0.02	10.5 ± 0.5	5.7 ± 0.3
16-WS-S	63 ± 1.2	0.17 ± 0.00	99.4 ± 0.02	9.7 ± 0.4	6.2 ± 0.2
17-SW-K	60 ± 0.9	0.22 ± 0.04	99.0 ± 0.05	10.5 ± 0.5	5.7 ± 0.2
18-HW-S	55 ± 0.6	0.26 ± 0.01	99.2 ± 0.02	10.4 ± 1.0	5.8 ± 0.4
19-HW-K	50 ± 0.8	0.19 ± 0.01	98.5 ± 0.02	10.1 ± 0.8	6.0 ± 0.4
ASTM	N/A	0.33–0.39 [40]	90 Min [41]	<257 [42]	-

Note: CS = corn stover, SW = softwood, HW = hardwood, WS = wheat straw, E = enzymatic hydrolysis, K = kraft, O = organosolv, S = soda, and L = lignosulfonate. ASTM = ASTM International, American Society for Testing and Materials. R-Value = measure of material’s ability to reduce heat flow.

**Table 4:** Foam formulations.

Raw Materials (g)	Control Foam without Lignin	30 wt.% Polyol Substitution with Lignin
Polyol	15	10.5
Lignin	0	4.5
Viscosity Reducer	1.5	1.5
Water	0.04	0.04
Surfactant	0.15	0.15
Catalysts	0.76	0.76
Flame Retardant	1.5	1.5
Blowing Agent	3.3	3.3
Isocyanate	28.79	28.79

## Data Availability

Not applicable.

## Sample Availability

Not available.
